# Teaching Research Methods and Statistics in eLearning Environments: Pedagogy, Practical Examples, and Possible Futures

**DOI:** 10.3389/fpsyg.2016.00339

**Published:** 2016-03-10

**Authors:** Adam J. Rock, William L. Coventry, Methuen I. Morgan, Natasha M. Loi

**Affiliations:** School of Behavioural, Cognitive, and Social Sciences, University of New England, ArmidaleNSW, Australia

**Keywords:** eLearning, pedagogy, research methods and statistics, *Second Life*, virtual worlds

## Abstract

Generally, academic psychologists are mindful of the fact that, for many students, the study of research methods and statistics is anxiety provoking (Gal et al., 1997). Given the ubiquitous and distributed nature of eLearning systems (Nof et al., 2015), teachers of research methods and statistics need to cultivate an understanding of how to effectively use eLearning tools to inspire psychology students to learn. Consequently, the aim of the present paper is to discuss critically how using eLearning systems might engage psychology students in research methods and statistics. First, we critically appraise definitions of eLearning. Second, we examine numerous important pedagogical principles associated with effectively teaching research methods and statistics using eLearning systems. Subsequently, we provide practical examples of our own eLearning-based class activities designed to engage psychology students to learn statistical concepts such as Factor Analysis and Discriminant Function Analysis. Finally, we discuss general trends in eLearning and possible futures that are pertinent to teachers of research methods and statistics in psychology.

## Introduction

Generally, academic psychologists are aware of students’ perceptions regarding the “dull, difficult, and distressing” nature of research methods and statistics (Haslam and McGarty, 2014, p. 1). In fact, there is a substantial body of literature devoted to investigating the effect of research methods and statistics on students’ anxiety (e.g., Gal et al., 1997). Academic procrastination resulting from statistics anxiety has been linked to numerous variables including the importance of statistics, anxiety associated with interpreting statistical results, anxiety related to exam and classroom contexts, fear of the statistics lecturer, and fear of asking for assistance (Onwuegbuzie, 2004). Importantly, various studies have suggested that effective teaching practices for reducing students’ statistics anxiety include a humorous teaching approach, encouragement from the teacher, and the acknowledgment of anxiety coupled with the introduction of coping strategies (see Pan and Tang, 2004).

Cigdem and Topcu (2015) stated that eLearning has extended into most areas of education provision. Given the ubiquitous and distributed nature of eLearning systems (Nof et al., 2015), teachers of research methods and statistics need to be cognizant of how to effectively use eLearning technologies to engage psychology students.

The aim of the present paper is to discuss critically how the use of eLearning systems may facilitate the engagement of psychology students in research methods and statistics. First, we critically appraise definitions of eLearning. Second, we discuss numerous important pedagogical principles associated with effectively teaching research methods and statistics using eLearning systems. Subsequently, we provide practical examples of our own eLearning-based class activities designed to engage psychology students to learn statistical concepts. Finally, we examine general trends in eLearning and possible futures that are pertinent to teachers of research methods and statistics in psychology.

## What is eLearning?

Numerous scholars define eLearning as a variety of online technologies (e.g., *Second Life*) used to facilitate the acquisition of knowledge (e.g., Lorenzi et al., 2004; Asuncion et al., 2007). Others (e.g., Jamison, 2008) moved beyond this rudimentary definition and formulated a dichotomy consisting of *traditional eLearning* (e.g., reading static hypertext pages) and *non-traditional eLearning* [e.g., interactions with avatars in *virtual worlds* (VWs)]. Tripartite models that distinguish between *basic eLearning* (e.g., online pages with assessment), *interactive eLearning* (e.g., the use of multi-media), and *advanced eLearning* (e.g., VWs populated by avatars) arguably superseded this dichotomy (Chapman, 2010). However, definitions of eLearning will evolve as technology evolves. For example, one may envision a historical moment where universities exist solely in cyberspace. In this scenario, the qualifier ‘e’ in eLearning would become redundant because all learning would be eLearning and, thus, eLearning would be defined as the accumulation of knowledge (i.e., learning; Reber and Reber, 2001). The aforementioned key definitional elements may be synthesized to produce the following definition: eLearning may be defined as the use of online technologies ranging from reading non-interactive contents pages to interacting with avatars in VWs for the purpose of acquiring knowledge and skills.

However, the aforementioned definition is problematic for a number of reasons. First, this definition assumes *a priori* that static hypertext pages constitute the most rudimentary end of the spectrum of eLearning tools whereas VWs and avatars should be located at the most sophisticated end. However, the question is whether *virtual reality* (VR; e.g., the use of immersive head-sets, data-gloves), rather than VWs, constitutes the most technologically sophisticated eLearning tool to date. Second, this definition does not explicitly include *mobile learning* and, thus, the portable aspect of eLearning. Third, given that the term being defined is *eLearning*, it is appropriate that the aforementioned definition is student-centered and, thus, focused on the acquisition of knowledge and skills rather than teaching. However, eLearning is inextricably bound with underlying pedagogical principles and, thus, any comprehensive definition of eLearning should contain an explicit reference to pedagogy. For instance, the social constructivist model was a key element of Tavangarian et al.’s (2004) definition of eLearning. Thus, it is noteworthy that the aforementioned definition of eLearning is bereft of a reference to pedagogy. Finally, based on an analysis of research articles and a survey of 43 persons, it appears that disparity exists regarding definitions applied to terms such as eLearning (Moore et al., 2011). For example, Moore et al. highlighted that there is disagreement regarding whether definitions of eLearning should be restricted to web-based technological tools (e.g., Nichols, 2003) or include interactive TV and satellite broadcasts (e.g., Ellis, 2004). Thus, from Ellis’ perspective, our aforementioned eLearning definition, with its focus on online technologies, is too restrictive. However, Monahan et al. (2008) argued that eLearning once only referred to learning delivered via electronic means. Importantly, with the inception of the internet, the definition of eLearning expanded and now encompasses entire courses delivered online. Thus, perhaps Ellis’ position is somewhat archaic. Taking the aforementioned points into consideration, for the purpose of the present paper we will define eLearning as follows: the pedagogically driven use of mobile and non-mobile web-based technologies ranging from hypertext pages to avatar-populated VWs and virtual realities for the purpose of acquiring knowledge and skills.

## Pedagogical Principles Associated with Teaching Research Methods and Statistics within eLearning Systems

McLoughlin and Lee (2008a) argued that eLearning pedagogies in tertiary education are often constrained by learning management systems (e.g., Blackboard, Moodle) that simply replicate instructor- and textbook-centered approaches in an online environment. That is, pedagogies need to be developed that allow teachers and learners to actualize the potential of eLearning tools. Unfortunately, some teachers, who are enthusiastic about the notion of eLearning, may use new digital technologies irrespective of whether such technologies are pedagogically effective, or in the complete absence of pedagogical considerations (Beetham and Sharpe, 2007). Thus, the following caution from Hughes (2008, p. 438) is timely: “Technology, without the pedagogy can be a fetishised and empty learning and teaching experience – stylised but without substance or simply electronic information push.” Consequently, the aim of this section is to discuss various pedagogical principles, which are pertinent to the effective teaching of research methods and statistics within an eLearning environment.

### The Pedagogy 2.0 and Presence Principles

McLoughlin and Lee (2008b, p. 56) stated that, “Pedagogy 2.0 integrates Web 2.0 tools that support knowledge sharing, peer-to-peer networking, and access to a global audience with socio-constructivist learning approaches to facilitate greater learner autonomy, agency, and personalization.” A *social-constructivist* pedagogical approach conceptualizes students as active learners who construct knowledge through: (1) the lenses of their personal experience; and (2) interactions with their teachers and peers (Farkas, 2012). Thus, according to Farkas, the “sage on the stage” model (i.e., the omniscient lecturer as the focal point) is replaced by a learning *community* whereby teachers and learners *co-create* knowledge.

Pedagogy 2.0 is similar, at least in part, to *Presence Pedagogy*, a method of teaching and learning predicated on social constructivist principles (Bronack et al., 2008). More specifically, Presence Pedagogy advocates the following principles: (1) benefiting from the presence of others; (2) encouraging interaction and facilitating community; and (3) sharing resources (Sanders and Melton, 2010). This model is typically applied in a VWs setting, but it may also serve as a guiding philosophy in the context of online discussion forums (Bronack et al., 2008). Such forums allow students to develop online communities and social support networks whereby peers co-create knowledge and share resources. In addition, the forums allow teachers to facilitate students engaged in the social construction of knowledge. For example, in one of our research methods and statistics eLearning systems, an online forum discussion thread emerged whereby students created, and posted, memes to illustrate particular statistical concepts. One series of memes depicted the popular cultural figure Chuck Norris (i.e., an American martial artist and actor) and included the following catch-cries: (1) “Negative correlation: The more Chuck Norris wants to kill you…the less chance you have of living”; and (2) “Perfect Correlation: X = The amount of times Chuck Norris kicks you…Y = Bone fractures you sustain” (Wendy Robertson, personal communication, Thursday 26 March, 2015). Various memes from this discussion thread were incorporated into subsequent lectures. Thus, eLearning systems may facilitate a reciprocal relationship or self-perpetuating feedback-loop whereby the “sage-on-the-stage” (i.e., the lecturer) invokes popular cultural references to illustrate statistical concepts that, in turn, catalyze a network of students to socially construct knowledge (e.g., create memes) that, in turn, further catalyze the lecturer to incorporate the students’ memes into subsequent lectures, and so on.

### The Learning as Knowledge Creation Principle

Presence pedagogy, with its focus on interaction as a principal method of *co-creating knowledge*, evokes Hong and Sullivan’s (2009) principle of *knowledge creation* via collective effort and innovation-oriented approaches. Hong and Sullivan (2009, p. 615) proposed that learning be defined in terms of knowledge creation, a process in which innovation is highlighted as the principal *instructional design goal*. Within this process, individuals are still active participants in their own learning, however, the emphasis is on the “innovative process of inquiry” (Hong and Sullivan, 2009, p. 614) whereby “something new is created and the initial knowledge is either substantially enriched or significantly transformed during the process” (Paavola et al., 2002, p. 24).

Knowledge creation not only further enhances individual knowledge, but “advance[s] community knowledge as a public product” (Hong and Sullivan, 2009, p. 616) as learners work together to develop their learning in the context of a social process that is participatory (McLoughlin and Lee, 2007). Knowledge creation aims to propel beyond a traditionally teacher-focused system in which teachers impart information to passive, receptive students to a system in which students take a more active and constructive role in their own learning. Thus, the emphasis is on a process in which learners actively work to create (or innovate) a path from a problem to a solution (Amabile, 1983; Hong and Sullivan, 2009).

According to Anderson and Dron (2011), social constructivism endorses knowledge creation as a social process. The sociality of humans is emphasized in social constructivism with the recognition that learning is most productive when the environment encourages a multitude of different perspectives in addition to validation, social discussion, and real-world application (Anderson and Dron, 2011). Knowledge creation is, thus, grounded in this constructivist tradition with its focus on “meaningful…activities to support situated learning and knowing” (Hong and Sullivan, 2009, p. 615). The chief point of convergence for this particular principle, however, is the idea of innovative instruction when building knowledge creating communities.

In order for knowledge creation to be actualized as a new pedagogical strategy, instructional design must develop into “a more innovation-oriented approach” (Hong and Sullivan, 2009, p. 614). Thus, utilizing eLearning environments incorporating innovative technologies such as VWs could facilitate the objective of knowledge creation. In collaboration with the teacher, and rather than simply being passive recipients of requisite knowledge (Paavola et al., 2002), students’ statistical acumen can be honed in a *knowledge-creating community* (Hong and Sullivan, 2009) in which everyone can work together to increase understanding and feelings of efficacy. As previously noted, research has demonstrated that statistics anxiety is linked with feelings of apprehension, inadequacy, and concerns regarding ability to grasp statistical concepts (e.g., Onwuegbuzie and Wilson, 2003; Onwuegbuzie, 2004). This anxiety has consequences for student performance and relates to students’ perceptions regarding their likelihood of passing or failing. In a knowledge-building community, the teacher, together with students who possess a greater statistical aptitude, can scaffold those students who feel less confident in their ability. This advantageous reciprocal relationship immerses students in an environment in which, by working together, students share and reflect upon their existing knowledge and together create new knowledge.

In order to promote a knowledge creating community, a collaborative assessment task could be developed in which students work together to deepen their understanding of the statistical notion “*p* < 0.05.” The logic of null hypothesis significance testing is one that many students struggle to grasp early in their statistics education, so this exercise would provide a medium by which they could enhance their comprehension. Via a wiki delivered through the learning management system, students working in groups of four would each contribute up to 250 words discussing their current understanding of what *p* < 0.05 means to them. They would be encouraged to consider real world analogies in order to actualize this relatively abstract concept as something more concrete and applicable to their everyday experiences. Once all students have contributed their paragraph, as a group, they would work together to assess and discuss each other’s work and provide feedback, improving and building upon each other’s knowledge. In this way, the integration of newly created knowledge with existing knowledge occurs (Anderson and Dron, 2011). As Green et al. (2010) stated, the use of collaborative assessment has the potential to result in an *adaptive know-how* coupled with an *emergent know-that*, meaning that by working together, students share and reflect upon their own existing knowledge and together create new knowledge.

### The Pedagogy of Desire Principle

A *pedagogy of desire* focuses on neglected aspects of teaching and learning (e.g., joy, happiness, transgression) in order to catalyze the desire to teach and learn and, thus, produce teachers and learners who are imaginative, creative agents (Pignatelli, 1999; Zembylas, 2007, p. 340). This principle is particularly pertinent in light of the observation that for many students the prospect of studying research methods and statistics is “boring” or “terrifying” (Gal et al., 1997). Thus, if learners experience boredom or anxiety, then a teacher of statistics might consider promoting a pedagogy of desire that “…produces and seduces imaginations” rather than creating an environment “associated…with repression and coercion” (Zembylas, 2007, p. 332).

We are mindful that previous research demonstrates that humorous teaching strategies may reduce students’ statistics anxiety and promote positive affect (e.g., happiness; e.g., Schacht and Stewart, 1990). The following are two examples of this strategy. In a class demonstration devised by one of us the aim is to elucidate the relationship between the reliability (i.e., consistency) and validity (i.e., accuracy) of psychological tests (e.g., an intelligence or IQ test). This demonstration requires a teaching assistant to function as a volunteer. The teacher informs the volunteer that he or she has developed an innovative new method for measuring a person’s IQ. The teacher produces a tape measure and measures the circumference of the volunteer’s head. On three separate occasions the teacher demonstrates that the circumference is, for example, 24 inches. Thus, the teacher states, “Let us conclude that our volunteer’s IQ is 24.” Subsequently, the teacher asserts that, “My innovative measure of IQ is reliable because I obtained the same result on three separate occasions. However, my method is not valid because an inch is not a metric that is interchangeable with an intelligence quotient or IQ score. Thus, if a measure is reliable it does not necessarily follow that it is valid.”

In another class demonstration devised by one of us, the objective is to explicate an inferential statistical test referred to as a Pearson’s product-moment correlation, which measures the strength of the relationship between two variables. To illustrate the concept of a correlation, one of us invokes the character “Barney” from the American situational-comedy “How I Met Your Mother.” The episode in which Barney is outlining the relationship between being hot (i.e., aesthetically pleasing) and crazy is described. As a class, we discuss that Barney is arguing that: (1) the correlation is high (i.e., strong); and (2) the direction of the relationship is positive (i.e., as hotness increases so too does craziness). At this point in the proceedings, students often like to venture anecdotes of their own past romantic relationships with hot and crazy individuals.

Importantly, the aforementioned class demonstrations are typically delivered in an eLearning context, using, specifically, *Adobe Connect*, “a web communication system that provides organizations with web communication solutions for training, marketing, and online teaching and learning” (Karabulut and Correia, 2008, p. 483). The teacher hosts the ‘meeting’ and, importantly, the students do not require software. Instead, the teacher e-mails a link to the students, which allows one to join the session via the internet.

### The “Smooth” Space versus “Striated” Space Principle

Deleuze and Guattari (1987, p. 474) asserted that *striated* or *gridded space* denotes space created and perpetuated by the State apparatus, which is formal, structured and hierarchical (Bayne, 2004). Massumi (1987, p. xiii) stated that, “the closed equation of representation, x = x = not y (I = I = not you)” is illustrative of State thought. In contrast, the *smooth*, *rhizomatic space* of *nomad thought* is a “decentered system of points that can connect in any order and without hierarchy” (Murphy and Smith, 2001, p. 1). The term *rhizome* is derived from botany and refers to “a network…that grows horizontally and discontinuously by sending out runners.”

Bayne (2004) applied the concepts of the “smooth” and the “striated” to pedagogical cyberspace (e.g., eLearning systems). According to Bayne (2004, p. 302), the “‘e-learning system’ which, in defining itself as a space of containment, regulation and efficient progression, functions as a strongly striating element within pedagogical web space.” More specifically, we note that eLearning systems often exhibit a striated (i.e., hierarchical) *presentation structure*. For example, an eLearning systems homepage is likely to consist of a group of several elements (e.g., general subject information, study schedule and materials, assessment items, forums). Each element leads to other groups of elements. For example, the “general subject information” element may lead to a group of elements (e.g., welcome, contact, how to purchase statistical analysis software, frequently asked questions).

In addition, the content-area of statistics is hierarchical. For example, analysis of variance is an extension of the *t-*test, and multiple regression/correlation is an extension of bivariate regression/correlation (Aron and Aron, 1999). Consequently, week-by-week research methods and statistics study topics featured in eLearning systems will tend to reflect this hierarchical characteristic (e.g., the *t*-tests study topic is covered before the analysis of variance topic, which is a special extension of the *t*-test). Thus, approaches to teaching research methods and statistics allow one to engage with a striated pedagogical cyberspace in terms of both presentation structure and content.

In contrast, the online discussion forums of eLearning systems provide an opportunity for students to co-create, and traverse, rhizomatic pedagogical cyberspace. For example, as previously stated, in one of our research methods and statistics eLearning systems, students have used online discussion forums to create memes using popular cultural references (e.g., Chuck Norris) with the aim of elucidating statistical concepts. Each popular cultural reference may be conceptualized as a point of a decentered system, which may connect with other points in a multitude of ways without recourse to order or hierarchy (Murphy and Smith, 2001). For instance, in various memes, our students juxtaposed the Teletubbies (i.e., a children’s television program), Mr. Spock (i.e., a character from the science fiction television program and movies, “Star Trek”), and Chuck Norris with the aim of co-creating and sharing knowledge with peers.

### The “Lines of Flight” Principle

Deleuze and Guattari (1987) developed the notion of *lines of flight* to refer to escape routes from striated space. A line of flight allows a learner, in the context of a relation to one’s self, to cultivate a resistance to codes and powers (Deleuze, 1988) and, thus, be able to think otherwise. Essentially, lines of flight may be conceptualized as “…instances of thinking and acting ‘outside of the box,’ with a greater understanding of what the box is, how it works, and how we can break it open and perhaps transform it for the better” (Lerner, n.d., paragraph 1).

The notion of escape routes from striated space is reminiscent of Heidegger’s (1962) concept of *Dasein*, which may be defined as “Being in the world, characterized…in terms of affective relationships with surrounding people and objects” (Blackburn, 1994, p. 94). *Being-in-the-world* equates to inauthentic being on the grounds that our affective relationships to people or objects function to constrain our cognitions, behaviors, and so on. In order to transition from inauthentic to authentic being, one must escape the influence of the “web” of affective relationships by utilizing one’s creativity and volition (i.e., “thinking outside the box”; Heidegger, 1962).

An example that one of us devised with the aim of creating a line of flight within an eLearning system is concerned with the ontology of numbers. The teacher pours a carton of milk into a saucer, writes a cat’s name (e.g., “Felix”) on a slip of paper and, subsequently, places the paper in the saucer. The teacher says to the class: “Felix initially appeared quite dehydrated but now he seems replenished!” Students invariably laugh and the teacher asks what is humorous about this scenario. The students explain that writing a cat’s name on a piece of paper does not constitute a real cat. The teacher responds, “Yes!” The teacher suggests that the linguistic term (i.e., word) “cat” is a signifier that is referentially linked to an object (i.e., the signified) in the external world with whiskers, fur, a tail and a tendency to “meow.” In addition, the teacher asserts that:

Feeding milk to a linguistic term is an example of confusing the signifier with the signified. It would seem to follow that I have never seen a number and, in fact, do not know what a number is. Why? If I were to write, for example, “8” on the board, then this would constitute a symbol (i.e., the signifier) that is referentially linked to a number (i.e., the signified). However, to assert that “8” is a number is to confuse the signifier with the signified just like I confused the slip of paper with “Felix” written on it with the physical object in the external world.

This demonstration may be delivered via web-conferencing tools (e.g., Adobe Connect) and creates a line of flight by encouraging students to reflect critically on the nature, essence, and existence of numbers and, thus, statistics.

## Practical Examples Using Virtual Worlds

As previously stated, traditional eLearning is often reducible to a “network of static hypertext pages” (Brusilovsky, 1999, p. 19), thereby constraining the learner to engage in repetitive read and click functions (Jamison, 2011). What are needed are emerging eLearning tools that facilitate an innovative student-centered experience that is *interactive* and *immersive*. One eLearning tool that allows teachers to be innovative is a VW, which may be defined as “a computer-simulated persistent spatial environment that supports synchronous communication among multiple users who are represented by avatars” (Jung and Kang, 2010, p. 219). VWs include *ActiveWorlds*, *Forterra Systems*, and *Entropia* (Messinger et al., 2009). Currently, in education, the most popular and mature VW platform is *Second Life* (SL; Warburton, 2009).

**FIGURE 1:**
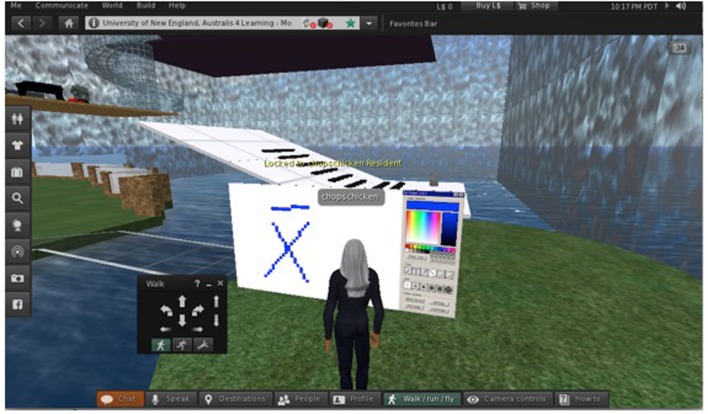
***Second Life* replicating traditional teaching methods via a virtual interactive whiteboard**.

The innovative potential of VWs provides an opportunity to reshape pedagogical approaches rather than merely replicate traditional teaching methods (Dreher et al., 2009). However, if one were to use SL to simply simulate a PowerPoint presentation in a lecture theater, then the potential for teaching innovation is neglected in favor of “static communication, a single presentation area, and multi-media integrated from Web 2.0 only” (p. 216); see **Figure 1.** Fundamentally, VWs allow the user to virtually experience an object or event rather than simply read text (Chow et al., 2007).

In comparison with the 2-D web, VWs provide numerous innovative ways to facilitate learning (Boulos et al., 2007). For instance, VWs may be used to provide simulated training with the aid of avatars (i.e., an online personal presence) and ‘bots’ (i.e., an online presence controlled by a machine rather than a human). Examples include role-play simulation in child psychiatry (Vallance et al., 2014), simulated pediatric dentistry (Papadopoulos et al., 2013), virtual patients teaching medical students communication skills (Stevens et al., 2006), and simulated medical emergencies designed to teach CPR to high school students (Youngblood et al., 2007). Numerous studies (e.g., Loftin and Kenney, 1995; Cohen et al., 2013) support the efficacy of simulated training.

In addition, VWs may be used to provide virtual field trips (VFTs), which, via web technologies, can simulate the experience of fieldwork (Arrowsmith et al., 2005). VFTs allow teachers and students to transcend the limitations of time, space, and finances. Garner and Gallo (2005) found no significant differences between a physical field trip group and a VFT group regarding student achievement. The authors concluded that activities such as these can and do promote learning.

Below are two practical examples of statistical methods that can be taught in an engaging and novel way using VWs. We chose VWs because previous research using a VW to engage students in research methods has shown promising results in improving student knowledge and confidence (Baglin et al., 2013). Our two examples focus on statistical tests that are typical of those taught at the 3rd/4th year university level in Psychology, and were chosen because, due to their complexity relative to other statistical methods taught at the same level, they are each better illustrated with a practical example. Providing practical examples in research methods and statistics can be a valuable method to assist students in understanding often abstruse concepts that are difficult to reconcile in the real-world. Research examining the utilization of practical and interesting examples in the teaching of statistics has found that students report a newfound enjoyment for the subject matter as well as seeing an increase in test scores (Burkley and Burkley, 2009). We have delivered these as live class activities for over four years, and the overwhelmingly positive feedback from students each year affirms they are an effective pedagogical resource.

### Practical Example One

#### Factor Analysis Lesson in a Virtual World such as *Second Life*

Factor analysis is a statistical method used to reduce a large number of variables to a smaller set that best capture the information in the original set. Variables that correlate highly are coalesced into one factor. If multiple factors emerge, then they are structured so as to be largely independent of one another (Cattell, 1952).

The following is a student demonstration designed to provide a rudimentary introduction to the concept of factor analysis in the context of *Second Life*.

(1) The teacher avatar (hereafter “teacher”) invites 15 to 20 student avatars (hereafter “students”) to stand at the front of the virtual class.
(2) The students are informed they each represent separate variables concerning hair color and, for simplicity, we are interested in the extent to which each variable (or student) correlates with the broader shades of either blonde or dark hair color.
(3) The teacher explains the goal of the demonstration is to reduce the number of variables from 20 to perhaps two or three.
(4) The teacher invites all students with blonde hair to stand together and all students with dark hair to stand together. In so doing, the factor analysis has derived just two factors (i.e., blonde and dark hair), and by using just these two factors the analysis captures a substantial amount of the variation in hair color that was present in the original 20 students. Clearly, having just two factors (or variables) is far more parsimonious than 20.
(5) There will be generally students with brown hair; these students were ignored until now. The teacher then asks whether these students should: (a) be combined with the blonde hair group to create a single blonde-brown group; (b) be combined with the dark hair group to create a single dark-brown group; or (c) form their own group. This allows the teacher to consider what number of factors would be ideal, two or three? The issue is central to factor analysis. For the sake of this demonstration, three factors may be selected.
(6) Typically, there is a student with red or gray hair in the virtual class. The teacher invites these students to walk to the front of the virtual class and join the group to which they belong. However, these students will fit into none of the existing groups. The teacher points out that these students represent an outlier at the variable level. These students are, accordingly, removed from the factor analysis and asked to sit down.
(7) The teacher explains that a factor is a composite of individual variables which all measure the same latent construct. In this example, we have an *amalgam* of various shades of brown that form a single brown hair factor. It is noted that there is a necessary loss of detail in the process of forming the factor. That is, each individual’s unique hair color is supplanted by the aggregate brown hair color.
(8) Finally, the teacher explains that these factors are used as predictors in subsequent analyses (e.g., predicting the dependent variable, ethnicity).

### Practical Example Two

#### Discriminant Function Analysis Lesson in a Virtual World such as *Second Life*

Discriminant Function Analysis (DFA) is a statistical method used to predict membership on a categorical (i.e., grouping) dependent variable (DV) from one or more continuous or binary independent variables (IVs). DFA is used when groups are known *a priori*. Thus, the output shows, for each group, the frequencies of the predicted group membership against the actual group membership in order to present intuitively, the prediction accuracy of the analysis (Cohen et al., 2003).

The following is a class activity designed to provide an illustration of DFA in the context of *Second Life*.

**FIGURE 2:**
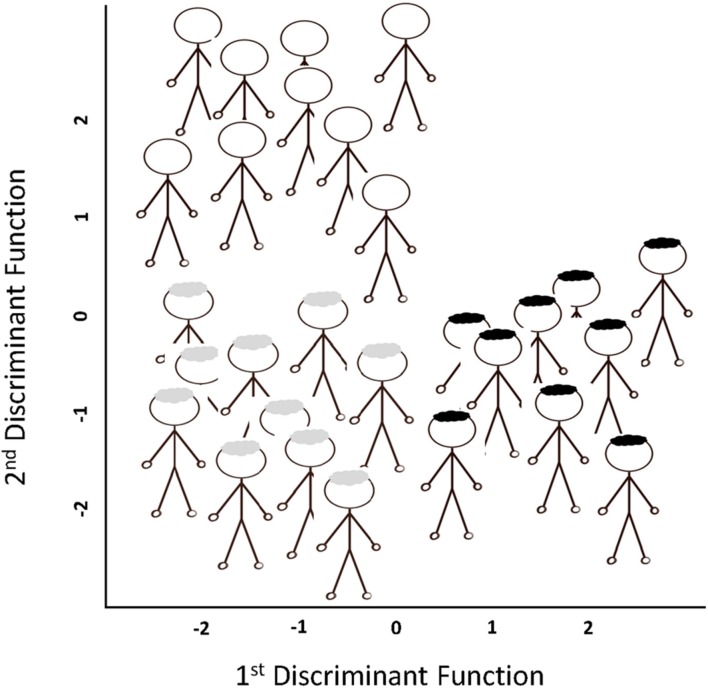
**A bird’s-eye view of the floor plan for the virtual class demonstration of a Discriminant Function Analysis (DFA).** The axes contain standardized values for each discriminant function. The class participants are presented here according to their father’s hair type as predicted by the DFA; that is, being bald (at the top), blonde (bottom left), or dark (bottom right).

(1) The teacher avatar (hereafter “teacher”) invites 15–30 student avatars (hereafter “students”) to line up in a virtual open space. (One may use between one and three lines depending on the number of students and the size of the virtual space.)
(2) For each line, the teacher nominates a student to be the DFA method “in action.”
(3) The teacher invites the nominated students to try and predict, for each student in their line, if each student’s father has dark, blonde, or no hair. Fathers’ hair type is the DV (i.e., grouping variable).
(4) The teacher explains that the predictions are based on multiple continuous IVs, which include each student’s hair color, complexion, and number of hair follicles. Clearly, not all predictions will be correct, which provides a useful illustration of the potential (in) accuracy of the model.
(5) The students are invited to stand in one of three groups that represent whether their father has (or had): (a) dark hair; (b) blonde hair; or (c) is bald. The location of each group is illustrated in **Figure 2.**
(6) Subsequently, the teacher explains that the angle of the first discriminant function, as shown in **Figure 2**, can differentiate between students with: (a) fathers with dark hair from (b) those with either blonde hair or no hair. The variables with a high loading on this function would be student complexion and student hair color.
(7) The teacher explains that the second discriminant function, as shown in **Figure 2**, differentiates (a) the bald group from (b) fathers with hair (dark or blonde). The variable loading high on this function would be the student’s number of hair follicles.
(8) The teacher highlights that the two discriminant functions are orthogonal to each other. If there were a third discriminant function it would point directly up in the air.
(9) The teacher reports that the scores on the discriminant functions represent standardized *z* scores, with the mean of zero being in approximately the middle, and high scores being above this and positive, and low scores being below this and negative, as shown in **Figure 2.**
(10) The teacher provides an example of what a standardized value on, for example, the first discriminant function represents. That is, if a student had a high score (i.e., greater than zero) on the first discriminant function they would be in the dark haired group. Importantly, however, if a student had a low score on this first discriminant function they could be in either the blonde or bald group. The teacher explains that it is only by also looking at the student’s score on the second discriminant function that we can discern which group they belong to. If a student had a low (i.e., less than zero), rather than high, score on the second discriminant function, and a low score on the first, they would be in the blonde group.
(11) Thus far, we have only attempted to predict group membership. When creating our model, we also need to assess the accuracy of the model by comparing our predictions against the true, rather than predicted, status. We can acquire this information by simply asking each student if his or her father is dark, blonde or bald.
(12) The teacher invites the students who were incorrectly classified to sit down.
(13) Within one group, the teacher explains that the students standing represent the accurate classifications of that group, which can be converted to the percentage correct. This step may be repeated for each of the remaining groups.
(14) Subsequently, the previous step is repeated for all students participating in the demonstration (i.e., the analysis). Thus, the students standing represent the total number of accurate classifications, which can be converted to the total percentage correct.
(15) Finally, the teacher states that DFA models are created with data where the true, rather than predicted, status is known. The goal of DFA is to use the model to generalize beyond the sample in order to predict group status for cases where the true state is unknown. To illustrate this point, the teacher pretends they are an orphan and do not know, or will ever know, their father’s identity. However, it is possible to use the DFA model to predict what group the teacher will fall into and, thus, the teacher’s father’s hair color.

## Current Trends in eLearning and Potential Futures

Martin et al. (2011) analyzed eLearning trends from 2004 to 2014 and identified two themes which we consider pertinent to teaching research methods and statistics:

(1) Mobile devices and the social web are the most important eLearning tools for the near future; and
(2) Games should be deemed an important eLearning tool.

These findings are supported by numerous studies (e.g., Arora et al., 2014; Bhalla, 2014; Yu et al., 2014).

Trend (1) refers to the current shift from eLearning to mobile learning (mLearning), which involves “the use of mobile or wireless devices for the purpose of learning while on the move” (Park, 2011, p. 79). An example of a mobile learning technology that pertains to teaching and learning research methods and statistics is StatHand, an application designed to help students cultivate statistical proficiency (About StatHand, 2015; see also Allen et al., 2015). We note that our students have reported using mobile learning devices while engaged in other activities such as horse riding and operating farm machinery (e.g., tractors, harvesters). Such experiences are characterized, in part, by multi-tasking and, thus, divided attention. Fittingly, Lahiri and Moseley (2012, p. 11) cautioned that the use of mobile devices as eLearning tools needs to be underpinned by pedagogical principles and an evidence-base otherwise the use of such tools “might lead to frustration, inequity, shallow learning, and distraction from the main purpose of enhancing learning and making students’ competent professionals.” Thus, in order to reduce students’ statistics anxiety and facilitate student engagement, teachers may wish to consider carefully how to effectively use mobile devices as part of the learning process, which may include the adoption of multiple hitherto unrealized pedagogical strategies (Yu et al., 2014).

Trend (2) refers to the realization by eLearning providers that video game technology can be used to develop fun and immersive simulations (Bhalla, 2014). It is noteworthy that a meta-analysis of game-based learning found that 34 of 65 studies reported statistically significant positive learning effects and only one study reported that computer games were less effective than conventional instruction (Ke, 2009). In addition, a more recent meta-analysis found that, when instructional support was provided, game-based learning enhanced the acquisition of knowledge and skills (Wouters and van Oostendorp, 2013). Trend (2) relates to the goal of facilitating student engagement with statistical concepts. In order to achieve this goal in the context of trend (2), teachers of research methods and statistics need to cultivate an understanding of how video game technologies and principles might be applied in their class. For example, a key principle underpinning the development of video game technology is the facilitation of states of “flow” in the user (i.e., being in the “zone”; Squire, 2003; Cowley et al., 2008; Annetta et al., 2009). If the video game is either too easy or too difficult the user will shift from a flow state to an ordinary waking state characterized by boredom or frustration, respectively (Jamison, personal communication, October 12, 2014). In this regard, we note that in our research methods and statistics computer labs, the proficiencies of students typically fall into three categories: novice, intermediate, and advanced. We have observed that the intermediate students tend to exhibit a flow state. In contrast, the advanced students consider the class too easy and are, thus, bored whereas the novice students regard the class as too difficult and are, thus, anxious and perhaps frustrated. Consequently, the challenge for teachers is to attempt to facilitate flow states in the novice and advanced students. In our own teaching, we have addressed this issue of discrepant learners by delivering separate classes for novice, intermediate, and advanced students. However, we acknowledge the practical issues (e.g., increase in academic workload) associated with such an undertaking. Nonetheless, in the context of using eLearning tools to facilitate student engagement with statistics one would be advised to develop tasks designed to optimize the flow states of learners.

## Conclusion

The objective of the present paper was to examine critically how teachers seeking to engage psychology students in research methods and statistics might use eLearning systems. We demonstrated how various eLearning-related pedagogical principles (i.e., Pedagogy 2.0, Presence Pedagogy, learning as knowledge creation, a pedagogy of desire, striated space versus rhizomatic space, lines of flight) might be applied in the context of teaching research methods and statistics, using examples from our own teaching. Subsequently, we devised two practical examples concerning how Virtual Worlds (e.g., *Second Life*) might be used to deliver class demonstrations concerning two advanced research methods, Factor Analysis and DFA. Finally, we discussed the relevance of mobile learning and video game principles (i.e., the effect of task difficulty on the flow states of the user) to student engagement with research methods and statistics.

In the current era of academic capitalism, which is characterized by the emergence of the entrepreneurial, online university, we note that teachers are constrained to engage in market-like behavior (Slaughter and Leslie, 1997) and provide consumers with *anywhere/anytime learning* (Twining, 2009). Thus, teachers are required to move beyond the notion of the traditional classroom with its face-to-face mode of delivery. In addition, the impending obsolescence of basic eLearning (e.g., students reading static hypertext pages) due to rapid developments in advanced eLearning (e.g., VWs populated by avatars; Chapman, 2010), has resulted in the need for teachers to engage in life-long learning with the aim of maintaining competence in the use of ever-changing eLearning tools and systems. However, we emphasize that the effective use of eLearning tools may be unlikely in the absence of the development of corresponding pedagogies (Hughes, 2008).

## Author Contributions

All authors listed, have made substantial, direct and intellectual contribution to the work, and approved it for publication.

## Conflict of Interest Statement

The authors declare that the research was conducted in the absence of any commercial or financial relationships that could be construed as a potential conflict of interest.

## References

[B1] About StatHand. (2015). Available at: https://www.stathand.net/Home/About

[B2] Allen P. Roberts L. Baughman F. van Rooy D. Rock A. Loxton N. (2015). *StatHand [Computer Software].* Sydney: Office for Learning and Teaching Available at: https://www.stathand.net/

[B3] Amabile T. M. (1983). The social psychology of creativity: a componential conceptualization. *J. Pers. Soc. Psychol.* 45 357–376. 10.1037/0022-3514.45.2.357

[B4] Anderson T. Dron J. (2011). Three generations of distance education pedagogy. *Int. Rev. Res. Open Distance Learn.* 12 80–97.

[B5] Annetta L. A. Minogue J. Holmes S. Y. Cheng M. T. (2009). Investigating the impact of video games on high school students’ engagement and learning about genetics. *Comput. Educ.* 53 74–85. 10.1016/j.compedu.2008.12.020

[B6] Aron A. Aron E. N. (1999). *Statistics for Psychology*, 2nd Edn Upper Saddle River, NJ: Prentice-Hall.

[B7] Arora C. Kaur C. Gupta A. Bhardawaj A. (2014). A review of recent E-learning trends: implementation and cognitive styles. *Int. J. Inform. Comput. Technol.* 4 215–220.

[B8] Arrowsmith C. Counihan A. McGreevy D. (2005). Development of a multi-scaled virtual field trip for the teaching and learning of geospatial science. *Int. J. Educ. Dev. Inform. Commun. Technol.* 1 42–56.

[B9] Asuncion J. V. Fichten C. Barile M. (2007). Which forms of eLearning are accessible to Canadian postsecondary students with disabilities? *Communiqué* 7:36.

[B10] Baglin J. Reece J. Bulmer M. Di Benedetto M. (2013). “Stimulating the data investigative cycle in less than two hours: using a virtual human population, cloud collaboration and a statistical package to engage students in a quantitative research methods course,” in *Proceedings of the Joint IASE/IAOS Satellite Conference*, eds Forbes S. Phillips Macao B..

[B11] Bayne S. (2004). Smoothness and striation in digital learning spaces. *E Learn. Digital Media* 1 302–316. 10.2304/elea.2004.1.2.6

[B12] Beetham H. Sharpe R. (eds) (2007). *Rethinking Pedagogy for a Digital Age. Designing and Delivering e-Learning.* London: Routledge.

[B13] Bhalla S. (2014). E-learning: tools, techniques and trends. *Int. J. Eng. Sci. Invent. Res. Dev.* 1 82–87.

[B14] Blackburn S. (1994). *Oxford Dictionary of Philosophy.* Oxford: Oxford University Press.

[B15] Boulos M. N. K. Hetherington L. Wheeler S. (2007). Second life: an overview of the potential of 3D virtual worlds in medical and health education. *Health Inform. Lib. J.* 24 233–245. 10.1111/j.1471-1842.2007.00733.x 18005298

[B16] Bronack S. Sanders R. Cheney A. Riedl R. Tashner J. Matzen N. (2008). Presence pedagogy: teaching and learning in a 3D virtual immersive world. *Int. J. Teach. Learn. Higher Educ.* 20 59–69.

[B17] Brusilovsky P. (1999). “Adaptive and intelligent technologies for web-based education,” in *Kunstliche Intelligenz, Special Issue on Intelligent Systems and Teleteaching* Vol. 4 eds Rollinger C. Peylo C. (Cambridge: Academic Press), 19–25.

[B18] Burkley E. Burkley M. (2009). Mythbusters: a tool for teaching research methods in psychology. *Teach. Psychol.* 36 179–184. 10.1080/00986280902739586

[B19] Cattell R. B. (1952). *Factor Analysis, for the Psychologist and Social Scientist.* New York, NY: Harper.

[B20] Chapman B. (2010). *How Long Does it take to Create learning?* Available at: http://www.chapmanalliance.com/howlong/

[B21] Chow A. Andrews S. Trueman R. (2007). “‘A ‘second life’: can this online, virtual reality world be used to increase the overall quality of learning and instruction in graduate distance learning programs?,” in *Proceedings of the Association for Educational Communications and Technology International Convention*, ed. Simonson M. (Bloomington, IN: Association for Educational Communications and Technology), 75–83.

[B22] Cigdem H. Topcu A. (2015). Predictors of instructors’ behavioral intention to use learning management system: a Turkish vocational college example. *Comput. Hum. Behav.* 52 22–28. 10.1016/j.chb.2015.05.049

[B23] Cohen D. Sevdalis N. Taylor D. Kerr K. Heys M. Willett K. (2013). Emergency preparedness in the 21st century: training and preparation modules in virtual environments. *Resuscitation* 84 78–84. 10.1016/j.resuscitation.2012.05.014 22659598

[B24] Cohen J. Cohen P. West S. G. Aiken L. S. (2003). *Applied Multiple Regression/Correlation Analysis for the Behavioral Sciences*, 3rd Edn Mahwah, NJ: Lawrence Erlbaum.

[B25] Cowley B. Charles D. Black M. Hickey R. (2008). Toward an understanding of flow in video games. *Comput. Entertain.* 6:20 10.1145/1371216.1371223

[B26] Deleuze G. (1988). *Foucault*, Trans. Hand S. Minneapolis, MN: University of Minnesota Press.

[B27] Deleuze G. Guattari F. (1987). *A Thousand Plateaus: Capitalism and Schizophrenia.* Minneapolis, MN: University of Minnesota Press.

[B28] Dreher C. Reiners T. Dreher H. Dreher N. (2009). Virtual worlds as a context suited for information systems education: discussion of pedagogical experience and curriculum design with reference to second life. *J. Inform. Syst. Educ.* 20 211–224.

[B29] Ellis R. (2004). *Down with Boring E-Learning!* (Interview with Dr. Michael W. Allen) Available at: http://www.astd.org/LC/2004/0704_allen.htm

[B30] Farkas M. (2012). Participatory technologies, pedagogy 2.0 and information literacy. *Library Hi Tech* 30 82–94. 10.1108/07378831211213229

[B31] Gal I. Ginsburg L. Schau C. (1997). “Monitoring attitudes and beliefs in statistics education,” in *The Assessment Challenge in Statistics Education*, eds Gal I. Garfield J. B. (Netherlands: IOS), 37–51.

[B32] Garner L. C. Gallo M. A. (2005). Field trips and their effect on student achievement and attitudes: a comparison of physical versus virtual field trips to the Indian River Lagoon. *J. Coll. Sci. Teach.* 34 14–17.

[B33] Green N. C. Edwards H. Wolodko B. Stewart C. Brooks M. Littledyke R. (2010). Reconceptualising higher education pedagogy in online learning. *Distance Educ.* 31 257–273. 10.1080/01587919.2010.513951

[B34] Haslam S. A. McGarty C. (2014). *Research Methods and Statistics in Psychology.* London: Sage Publisher.

[B35] Heidegger M. (1962). *Being and Time*, Trans. Macquarrie J. Robinson E. (New York, NY: Harper & Row).

[B36] Hong H.-Y. Sullivan F. R. (2009). Towards an idea-centred, principle-based design approach to support learning as knowledge creation. *Educ. Technol. Res. Dev.* 57 613–627. 10.1007/s11423-009-9122-0

[B37] Hughes J. (2008). “Letting in the Trojan mouse: using an eportfolio system to re-think pedagogy,” in *Proceedings of Ascilite, Hello! Where are you in the Landscape of Educational Technology?*, Melbourne Available at: http://www.ascilite.org.au/conferences/melbourne08/procs/hughes.pdf

[B38] Jamison J. B. (2008). *Educators in a Strange Land: The Experience of Traditional Educators When Immersed into the Virtual Environment of Second Life.* Doctoral dissertation, ProQuest Dissertations and Theses database, UMI No.3307549 Capella University, Minneapolis, MN.

[B39] Jamison J. B. (2011). *Trance Formational Learning [PowerPoint slides].* Available at: https://prezi.com/zsjq5tdoja6k/tranceformational-learning/

[B40] Jung Y. Kang H. (2010). User goals in social virtual worlds: a means-end chain approach. *Comput. Hum. Behav.* 26 218–225. 10.1016/j.chb.2009.10.002

[B41] Karabulut A. Correia A. (2008). “Skype, Elluminate, Adobe Connect, Ivisit: a comparison of web-based video conferencing systems for learning and teaching,” in *Proceedings of Society for Information Technology & Teacher Education International Conference 2008*, eds McFerrin K. Weber R. Carlsen R. Willis D. (Chesapeake, VA: Association for the Advancement of Computing in Education), 481–484.

[B42] Ke F. (2009). “A qualitative meta-analysis of computer games as learning tools,” in *Handbook of Research on Effective Electronic Gaming in Education*, ed. Ferdig R. E. (New York, NY: Information Science Reference), 1–32.

[B43] Lahiri M. Moseley J. L. (2012). Is mobile learning the future of 21st century education? Educational considerations from various perspectives. *Educ. Technol.* 52 3–13.

[B44] Lerner J. (n.d.). *Lines of Flight.* Available at: http://www.linesofflight.net/linesofflight.htm (accessed April 3, 2007)

[B45] Loftin R. B. Kenney P. (1995). Training the Hubble space telescope flight team. *IEEE Comput. Graph. Appl.* 15 31–37. 10.1109/38.403825

[B46] Lorenzi F. MacKeogh K. Fox S. (2004). Preparing students for learning in an online world: an evaluation of the Student Passport to eLearning (SPEL) model. *Eur. J. Open Distance Learn.* 1. Available at: http://www.eurodl.org/?p=archives&year=2004&halfyear=1&article=108

[B47] Martin S. Diaz G. Sancristobal E. Gil R. Castro M. Peire J. (2011). New technology trends in education: seven years of forecasts and convergence. *Comput. Educ.* 57 1893–1906. 10.1016/j.compedu.2011.04.003

[B48] Massumi B. (1987). “Translator’s foreword: pleasures of philosophy,” in *A Thousand Plateaus: Capitalism and Schizophrenia*, eds Deleuze G. Guattari F. (Minneapolis, MN: University of Minnesota Press), 9–15.

[B49] McLoughlin C. Lee M. J. (2008a). “Mapping the digital terrain: new media and social software as catalysts for pedagogical change,” in *Hello! Where are you in the Landscape of Educational technology? Proceedings of Ascilite*, Melbourne.

[B50] McLoughlin C. Lee M. J. (2008b). The three P’s of pedagogy for the networked society: personalization, participation, and productivity. *Int. J. Teach. Learn. Higher Educ.* 20 10–27.

[B51] McLoughlin C. Lee M. J. W. (2007). “Social software and participatory learning: pedagogical choices with technology affordances in the web 2.0 era,” in *Proceedings of Ascilite: ICT: Providing Choices for Learners and Learning*, Singapore, 664–675. Available at: http://www.ascilite.org.au/conferences/singapore07/procs/

[B52] Messinger P. R. Stroulia E. Lyones K. Bone M. Nu R. H. Smirnov K. (2009). Virtual worlds – Past, present and future: new directions in social computing. *Decis. Support Syst.* 47 204–228. 10.1016/j.dss.2009.02.014

[B53] Monahan T. McArdle G. Bertolotto M. (2008). Virtual reality for collaborative e-learning. *Comput. Educ.* 50 1339–1353. 10.1016/j.compedu.2006.12.008

[B54] Moore J. L. Dickson-Deane C. Galyen K. (2011). e-Learning, online learning, and distance learning environments: are they the same? *Int. Higher Educ.* 14 129–135. 10.1016/j.iheduc.2010.10.001

[B55] Murphy D. W. Smith T. S. (2001). What I hear is thinking too: deleuze and Guattari go pop. *Echo* 3 1–35.

[B56] Nichols M. (2003). A theory of eLearning. *Educ. Technol. Soc.* 6 1–10.

[B57] Nof S. Y. Ceroni J. Jeong W. Moghaddam M. (2015). *Revolutionizing Collaboration Through e-Work, e-Business, and e-Service.* Berlin: Springer.

[B58] Onwuegbuzie A. J. (2004). Academic procrastination and statistics anxiety. *Assess. Eval. Higher Educ.* 29 3–19. 10.1080/0260293042000160384

[B59] Onwuegbuzie A. J. Wilson V. A. (2003). Statistics anxiety: nature, etiology, antecedents, effects, and treatments – a comprehensive review of the literature. *Teach. Higher Educ.* 8 195–209. 10.1080/1356251032000052447

[B60] Paavola S. Lipponen L. Hakkarainen K. (2002). “Epistemological foundations for CSCL: a comparison of three models of innovative knowledge communities,” in *Computer-Supported Collaborative Learning: Foundations for a CSCL Community: Proceedings of the Computer-Supported Collaborative Learning 2002 Conference*, ed. Stahl G. (Hillsdale, NJ: Lawrence Erlbaum), 24–32.

[B61] Pan W. Tang M. (2004). Examining the effectiveness of innovative instructional methods on reducing statistics anxiety for graduate students in the social sciences. *J. Instruct. Psychol.* 31 149–159.

[B62] Papadopoulos L. Pentzou A. E. Louloudiadis K. Tsiatsos T. K. (2013). Design and evaluation of a simulation for pediatric dentistry in virtual worlds. *J. Med. Internet Res.* 15 806–811. 10.2196/jmir.2651 PMC3841347 24168820

[B63] Park Y. (2011). A pedagogical framework for mobile learning: categorizing educational applications of mobile technologies into four types. *Int. Rev. Res. Open Distance Learn.* 12 78–102.

[B64] Pignatelli F. (1999). Education and the subject of desire. *Rev. Educ.* 20 337–352. 10.1080/1071441980200404

[B65] Reber A. S. Reber E. (2001). *The Penguin Dictionary of Psychology*, 3rd Edn London: Penguin.

[B66] Sanders R. L. Melton S. J. (2010). The AETZone experience: a qualitative analysis of the use of presence pedagogy in a 3D immersive learning environment. *J. Learn. Teach.* 6 62–70.

[B67] Schacht S. Stewart B. J. (1990). What’s funny about statistics? A technique for reducing student anxiety. *Teach. Sociol.* 18 52–56. 10.2307/1318231

[B68] Slaughter S. Leslie L. L. (1997). *Academic Capitalism: Politics, Policies, and the Entrepreneurial University.* London: The John Hopkins University Press.

[B69] Squire K. (2003). Video games in education. *Int. J. Intel. Games Simulat.* 2 49–62.

[B70] Stevens A. Hernandez J. Johnsen K. Dickerson R. Raij A. Harrison C. (2006). The use of virtual patients to teach medical students history taking and communication skills. *Am. J. Surgery* 191 806–811. 10.1016/j.amjsurg.2006.03.002 16720154

[B71] Tavangarian D. Leypold M. E. Nölting K. Röser M. Voigt D. (2004). Is e-learning the solution for individual learning? *Electr. J. E-learn.* 2 273–280.

[B72] Twining P. (2009). Exploring the educational potential of virtual worlds – Some reflections from the SPP. *Br. J. Educ. Technol.* 40 496–514. 10.1111/j.1467-8535.2009.00963.x

[B73] Vallance A. K. Hemani A. Fernandez V. Livingstone D. McCusker K. Toro-Troconis M. (2014). Using virtual worlds for role play simulation in child psychiatry: an evaluation study. *Psychiatr. Bull.* 38 204–2010. 10.1192/pb.bp.113.044396 PMC4180983 25285217

[B74] Warburton S. (2009). Second Life in higher education: assessing the potential for and the barriers to deploying virtual worlds in learning and teaching. *Br. J. Educ. Psychol.* 40 414–426. 10.1111/j.1467-8535.2009.00952.x

[B75] Wouters P. van Oostendorp H. (2013). A meta-analytic review of the role of instructional support in game-based learning. *Comput. Educ.* 60 412–425. 10.1016/j.compedu.2012.07.018

[B76] Youngblood P. Hedman L. Creutzfeld J. Fellander-Tsai L. Stengard K. Hansen K. (2007). Virtual worlds for teaching the new CPR to high school students. *Stud. Health Technol. Inform.* 125 515–519. 17377340

[B77] Yu C. Lee S. J. Ewing C. (2014). “Mobile learning: emerging trends, issues, and challenges in teaching and learning,” in *Proceeding of the World Conference on E-Learning in Corporate, Government, Healthcare, and Higher Education*, ed. Bastiaens T. (Chesapeake, VA: Association for the Advancement of Computing in Education), 2126–2136.

[B78] Zembylas M. (2007). Risks and pleasures: a Deleuzo-Guattarian pedagogy of desire in education. *Br. Educ. Res. J.* 33 331–347. 10.1080/01411920701243602

